# 3D Porous Architecture of Stacks of β-TCP Granules Compared with That of Trabecular Bone: A microCT, Vector Analysis, and Compression Study

**DOI:** 10.3389/fendo.2015.00161

**Published:** 2015-10-12

**Authors:** Daniel Chappard, Lisa Terranova, Romain Mallet, Philippe Mercier

**Affiliations:** ^1^GEROM Groupe Etudes Remodelage Osseux et bioMatériaux – LHEA, IRIS-IBS Institut de Biologie en Santé, CHU d’Angers, L’Université Nantes Angers Le Mans, Angers, France; ^2^Service Commun d’Imagerie et Analyses Microscopiques (SCIAM), IRIS-IBS Institut de Biologie en Santé, CHU d’Angers, L’Université Nantes Angers Le Mans, Angers, France; ^3^Laboratoire d’Anatomie, Faculté de Médecine, Angers, France

**Keywords:** porosity, microCT, 3D packing, 3D geometry, bone graft, fractal, β-TCP, granules

## Abstract

The 3D arrangement of porous granular biomaterials usable to fill bone defects has received little study. Granular biomaterials occupy 3D space when packed together in a manner that creates a porosity suitable for the invasion of vascular and bone cells. Granules of beta-tricalcium phosphate (β-TCP) were prepared with either 12.5 or 25 g of β-TCP powder in the same volume of slurry. When the granules were placed in a test tube, this produced 3D stacks with a high (HP) or low porosity (LP), respectively. Stacks of granules mimic the filling of a bone defect by a surgeon. The aim of this study was to compare the porosity of stacks of β-TCP granules with that of cores of trabecular bone. Biomechanical compression tests were done on the granules stacks. Bone cylinders were prepared from calf tibia plateau, constituted high-density (HD) blocks. Low-density (LD) blocks were harvested from aged cadaver tibias. Microcomputed tomography was used on the β-TCP granule stacks and the trabecular bone cores to determine porosity and specific surface. A vector-projection algorithm was used to image porosity employing a frontal plane image, which was constructed line by line from all images of a microCT stack. Stacks of HP granules had porosity (75.3 ± 0.4%) and fractal lacunarity (0.043 ± 0.007) intermediate between that of HD (respectively 69.1 ± 6.4%, *p* < 0.05 and 0.087 ± 0.045, *p* < 0.05) and LD bones (respectively 88.8 ± 1.57% and 0.037 ± 0.014), but exhibited a higher surface density (5.56 ± 0.11 mm^2^/mm^3^ vs. 2.06 ± 0.26 for LD, *p* < 0.05). LP granular arrangements created large pores coexisting with dense areas of material. Frontal plane analysis evidenced a more regular arrangement of β-TCP granules than bone trabecule. Stacks of HP granules represent a scaffold that resembles trabecular bone in its porous microarchitecture.

## Introduction

Bone is a calcified connective tissue, which fulfils a large number of functions in the body (1). Being both rigid and elastic due to the unique composition of its matrix made of collagen type I and hydroxyapatite crystals, it is adapted to gravity and muscle contractions. Bone cells are able to remodel and adapt bone mass and architecture throughout life allowing repair of microdamage (microcracks, trabecular microfractures) or fracture. To reduce the amount of this material within the body, Nature has developed a number of strategies to provide the maximum biomechanical competence with a genetically controlled bone mass. During aging, epigenetic factors, such as gravity and muscle strains, also modify bone mass and microarchitecture (Wolff’s Law). Trabecular microarchitecture is now a well-recognized type of self-organization that fulfils this condition (2, 3).

Bone loss can occur systemically, for example, during aging. However, localized bone loss can also occur, as in some intra-bony defects, such as solitary or aneurysmal cysts, or acquired bone defects as observed after tooth extraction or during hip prosthesis revision. Granular biomaterials are often used to fill these defects, especially in non-bearing bones. Among the different orthophosphates that can be produced in large quantity by industry, beta-tricalcium phosphate (β-TCP) appears the most suitable, particularly in maxillo-facial and dental surgery (4). Granules of 1000–2000 μm diameter represent the most commonly used size for filling alveolar sockets or increasing bone volume by sinus lift (5). However, when the surgeons place granules within a bone defect, it is the voids between the granules that represent the interconnected space available for vascular sprouts and bone cells to invade the grafted area. Calcium phosphate ceramics are known to be too brittle to be placed in load-bearing areas. Ceramic granules allow a perfect filling of the bone defects, but they are limited to grafted areas where no significant strength is needed (alveolar ridge augmentation, sinus lift …) (5). Little is known about the 3D arrangement of a grafted stack of granules, and if the porosity of the stack is similar to that of trabecular bone to allow the accessibility of biological fluids, body fluids, cells, and vascularization within the grafted site (6, 7). In addition, when filling a bone defect, the surgeon has to avoid crushing of the granules to avoid altering the granules’ microarchitecture (8). Hence, the biomechanical resistance of the granules in compression is also an important factor to be taken into account when choosing the grafting material (9).

The aims of the present study were (1) to compare the pore microarchitecture of the two types of β-TCP granules providing stacks of low porosity (LP) and high porosity (HP), respectively, with that of the two types of trabecular bone [high density (HD) and low density (LD)], in order to assess the granular stack that had the pore microarchitecture more similar to trabecular bone; (2) to compare the biomechanical properties in compression of these two types of granules.”

## Materials and Methods

### Bone Samples

For the bone with HD, five bone cylinders were harvested from the upper tibia epiphysis of young butchered animals (calves, aged around 15 months, 500–600 kg). The calibrated cylinders (10 mm in diameter) were obtained with a diamond-tipped coring tool (Starlite Industries, Rosemont, PA, USA) perpendicular to the tibial plateau. Similarly, five cylinders (10 mm diameter) with low bone density (LD) were prepared from the upper tibial epiphysis from five aged human cadavers from the Anatomical Laboratory (mean age 78.3 ± 5.2 years). For both the HD and LD cylinders, the articular and subchondral bone were removed to provide cylinders 23–25 mm in length containing only the secondary spongiosa. The subjects had given their body to science before death and had completed a form indicating that their corpses were to be used for medical education and research. Subjects were examined upon arrival at the laboratory of anatomy of the Faculty of Medicine. All bone samples were harvested following a strict protocol of the laboratory of anatomy; e.g., it was impossible to know any detail which could lead to the subject’s identification (causes of death, previous medical records).

### β-TCP Granules

Beta-tricalcium phosphate granules were obtained from Kasios (L’Union, France). Granules were prepared by polyurethane foam technology and used as received. This technique has been extensively described elsewhere (10, 11). Two types of granules were used and placed in polyethylene test tubes (10 mm in diameter): compact granules were prepared with 25 g of β-TCP grain in a fixed volume of slurry and provided a LP stacks; lighter granules with 12.5 g of β-TCP grain 1 in the same volume of slurry produced the HP stacks (12).

The morphology of the two types of granules was qualitatively evaluated by scanning electron microscopy (SEM, Jeol JSM 6301F) before analysis. The β-TCP granules were glued on an aluminum stub with a double-sided carbon sellotape. They were gold-coated by sputtering with an MED 020 (Bal-Tec, Chatillon sur Cher, France). Images were captured at a 3-kV acceleration voltage in the secondary electron mode.

### Biomechanical Analysis

**Figure 1:**
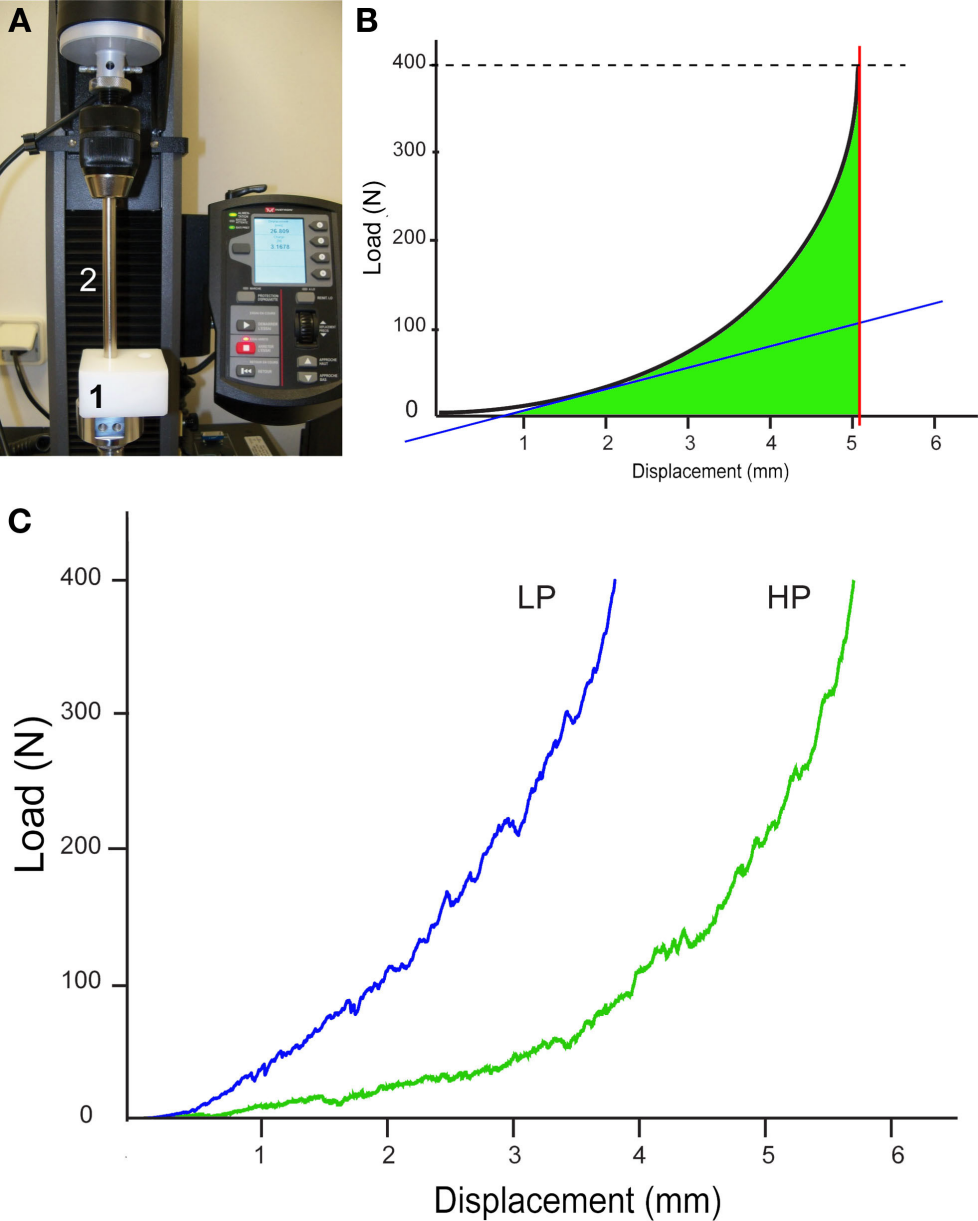
**Biomechanical analysis of the two types of stacks of granule (HP and LP)**. **(A)** Image of the compression machine; granules are placed in the cylindrical test cell (1) and compressed with the punch (2). **(B)** Theoretical load–displacement curve between displacement and force obtained by compression; the slope of the tangent to the curve is automatically provided (blue line), thus providing stiffness; the maximum displacement of the punch at 400 N is obtained by the red line; the area under the curve (in green) represents the work to failure. **(C)** The curves obtained for the HP and LP stacks of granules.

The compression characteristics of the two types of β-TCP granules were determined on an Instron 5942 machine with Bluehill software (Instron, Elancourt, France) (Figure 1A). As the test is destructive, new intact granules were placed into the cylindrical test cell of the machine and compacted with an adapted punch 10 mm in diameter. The maximum applied load was fixed at 400 N with a 2-mm min^−1^ displacement. For each type of stacks of granule (HP or LP), the load–displacement curve was recorded (N/mm) automatically by the machine. Stiffness was automatically provided by measuring the slope of the tangent to the curve at the beginning of the deformation (N/mm). The maximum displacement of the punch (in mm) was obtained when the load reached 400 N. The work to failure (N⋅mm) was determined as the area under the load–displacement curve from yield until failure and represents the energy absorbed by bone during plastic deformation (Figure 1B) (13). These parameters were calculated according to previously published equations (14, 15). Analysis was repeated five times for each type of granules and the averaged values were considered.

### MicroCT Analysis

MicroCT analysis was performed on a Skyscan 1172 microcomputed tomograph (Bruker microCT, Kontich, Belgium). Bone samples were placed in polystyrene test tubes (10 mm in diameter) and scanned while in fixative (10% formalin). Granules of β-TCP were placed in similar tubes and gently agitated to allow the granules to settle and optimize their 3D spatial arrangement in the dry state. Tubes were filled with β-TCP granules up to 30 mm in height. For each series of granules, analysis was done in triplicate. The microCT was operated at 80 kV, 100 μA with a 0.5-mm aluminum filter. The pixel size was fixed at 4.95 μm and the angular rotation step at 0.25°. 3D models of bones and granules were obtained with VG Studiomax (Volume Graphics GmbH, Heidelberg, Germany) operating in the volume rendering mode. Microarchitecture parameters were obtained with the CTAn software (Bruker) after global thresholding of the pores (16). They included measures of porosity (Po, in %), mean pore diameter (Po⋅Diam, in μm), and specific surface [representing the interface between bone or biomaterial and the porosity, BS/TV, in mm^2^/mm^3^, where BS is the whole pore surface and TV is the volume of interest according to the ASBMR nomenclature (17)]. For the bone cylinders and the granule stacks, the region of interest (ROI) over-imposed on the 2D binarized sections was a 5-mm wide square; the height of analysis of the samples has concerned 15 mm. Pore diameter was determined by the sphere-fitting method in the CTAn software (18). In order to further investigate the porosity, the binarized stacks of 2D microCT sections of each sample were analyzed by software written in Matlab (MathWorks, Natick, MA, USA). The algorithm for evaluation of porosity used a vector projection on a frontal plane (Vectopor) and has been extensively described elsewhere (19). In the present study, the pixels of the ROI which belonged to the same column received the same pseudo-color according to the number of pixels superimposed on pores. A look-up table (LUT) was designed ranging from deep blue (no porosity) to red (high porosity). Intermediate values depend on the value of porosity along the vector according to the LUT. The frontal plane vector projection image was assigned intensity values for each pixel based on the number of pixels, which contained pores in the related column of the microCT binarized image. The frontal plane image was saved with the colorized LUT and analyzed by the FracLac plugin developed for ImageJ (20, 21). The box-plot fractal dimension (*D*_f_) and the lacunarity (λ) of the frontal plane images were determined as previously described (19). The box-plot fractal dimension evaluates the complexity of the material to fill the reference space and is helpful in the study of porous objects, such as bone (22, 23) or materials (24, 25). Lacunarity is another recently described fractal parameter reported to improve the description of a fractal porous object (26, 27). Lacunarity is influenced by the variation of the pores in a given structure; a low lacunarity reflects homogeneity, while a high lacunarity is an indicator of heterogeneity (28, 29).

### Statistical Analysis

Statistical analysis was done with Systat 13 (Systat Inc., San José, CA, USA). For each parameter, results are expressed as mean ± SD. Differences for porosity between the four groups (HD, LD, HP, and LP) were sought with the Kruskal–Wallis non-parametric analysis of variance followed by the Conover–Inman test for all pairwise comparisons. For biomechanical parameters, differences between the HP vs. LP groups were analyzed by the non-parametric Mann–Whitney *U* test. A difference was considered as significant when *p* < 0.05.

## Results

### Qualitative Examination of the β-TCP Granules

**Figure 2:**
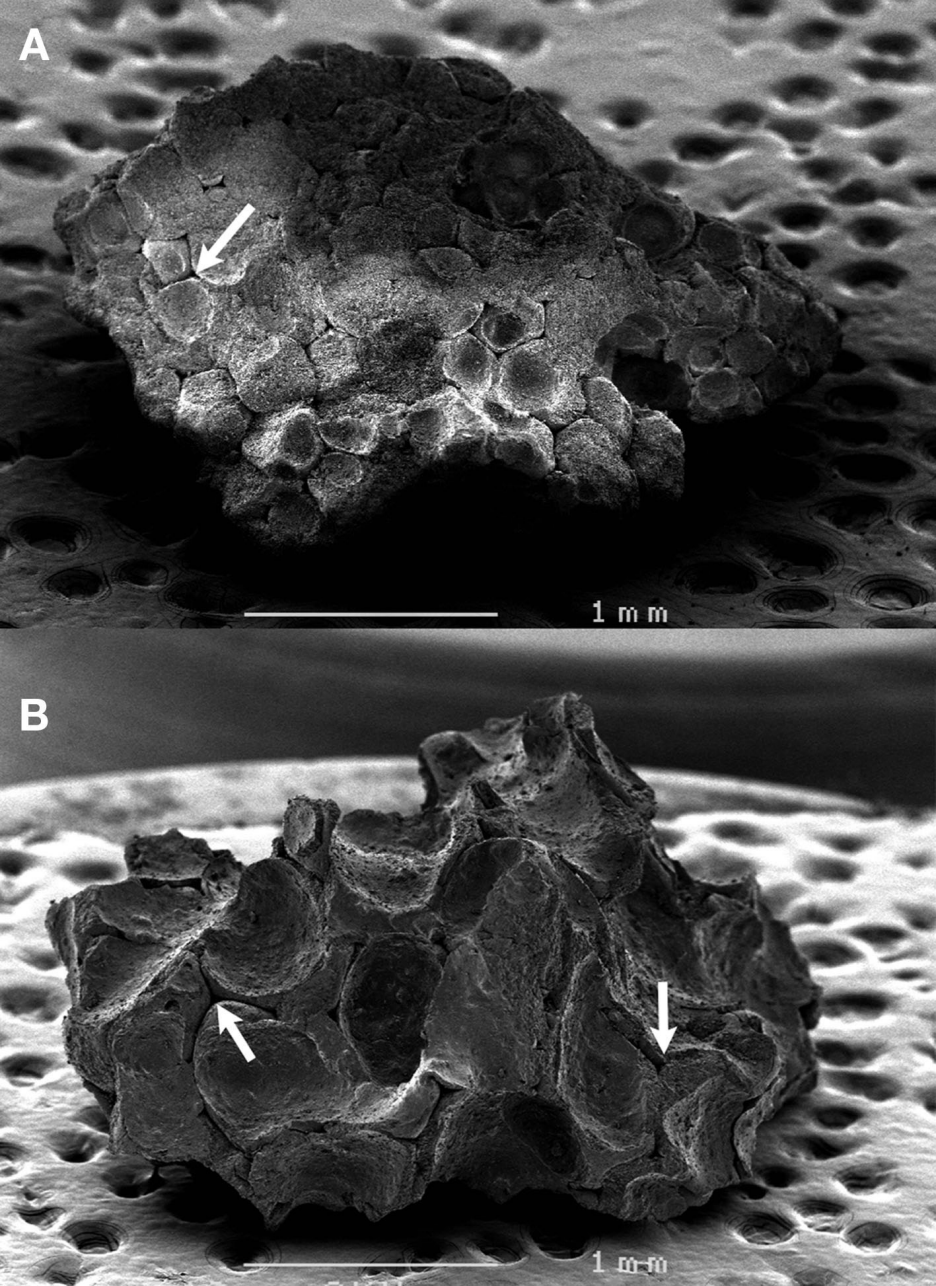
**SEM analysis of the two types of **β**-TCP granules**. **(A)** LP granules prepared with 25 g in the slurry, **(B)** HP granules prepared with 12.5 g. Note the inner porosity due to the sublimation of the polyurethane foam as triangular voids connected by thin channels (arrows).

The difference in morphology of the granules was evident to the naked eye and confirmed by SEM. The 12.5-g preparation of granules was the most porous with thin walls of sintered β-TCP, while the 25-g granule preparation was denser, with numerous concave surfaces and occasional macropores (Figure 2). In all cases, the inner porosity due to sublimation of the polyurethane foam was clearly identified as triangular voids connected by thin channels.

### Biomechanical Analysis of the β-TCP Granules

The load vs. displacement curves of both HP and LP types of granules are shown in Figure 1C. The first domain of the curve corresponds to the collapse of the granules, after which the deformation is due to their irreversible compression. The HP stack had a statistically significant higher maximum displacement compared to the LP stack, as expected (*p* < 0.05, Table 1). On the contrary, the stiffness and the work to failure were significantly higher in the LP stacks compared to the HP stack (*p* < 0.05 in both cases).

**Table 1:** **Morphometric parameters of bone cylinders with a high-density (HD), low-density (LD), and β-TCP granules (compact low porosity granules: LP; high porosity granules: HP)**.

	Bone		Granules	
	HD	LD	LP	HP
Po (%)	69.1 ± 6.4	88.8 ± 1.5*	58.9 ± 1.3*	75.3 ± 0.4*^§^
BS/TV (mm^2^/mm^3^)	4.08 ± 1.05	2.06 ± 0.26*	4.98 ± 0.09	5.56 ± 0.11^§^
Po⋅Diam (μm)	458 ± 123	875 ± 112*	359 ± 7	321 ± 7^§^
Fractal dimension *D*_f_	2.747 ± 0.017	2.762 ± 0.014	2.635 ± 0.034	2.749 ± 0.009
Lacunarity (λ)	0.087 ± 0.045	0.037 ± 0.014*^,§^	0.220 ± 0.110	0.043 ± 0.007^§^
Stiffness (N/mm)			65.4 ± 4.5	14.4 ± 1.8^§^
Max. displac. (mm)			3.90 ± 0.03	5.30 ± 0.31^§^
Work to failure (N⋅mm)			487 ± 7	447 ± 26^§^

*Data are provided as mean ± SD. Differences between the four groups were sought with the Kruskal–Wallis analysis of variance followed by the Conover–Inman post hoc test for all pairwise comparisons. For biomechanical parameters, differences between the HP vs. LP groups were analyzed by the Mann–Whitney U test*.

*Significantly different from HD cylinders with p < 0.05.

*^§^Significantly different from LP granules with p < 0.05*.

### Morphometric Analysis of Bone and Granules

**Figure 3:**
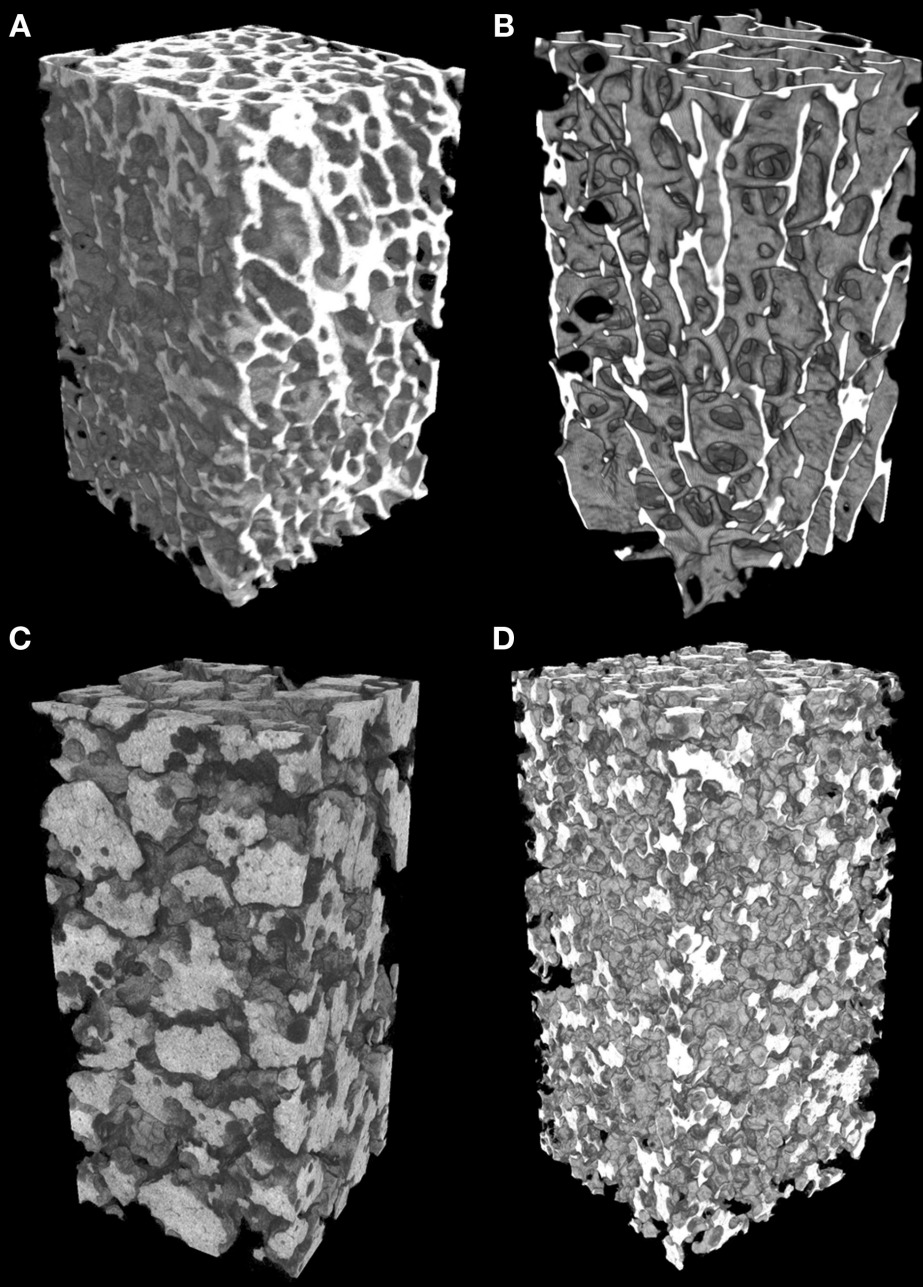
**Microtomographic analysis of the different types of materials (appearing in white)**. **(A)** High-density bone (HD) with numerous trabeculae and low porosity; **(B)** low-density bone (LD) from aged subjects with reduced trabeculae and increased porosity; **(C)** of compact granules (LP) of β-TCP; **(D)** scaffold of HP granules. All images have been prepared with a square ROI of 5 mm side; so the volume of interest is a parallelepiped (height: 15 mm; square section: 5 mm).

**Figure 4:**
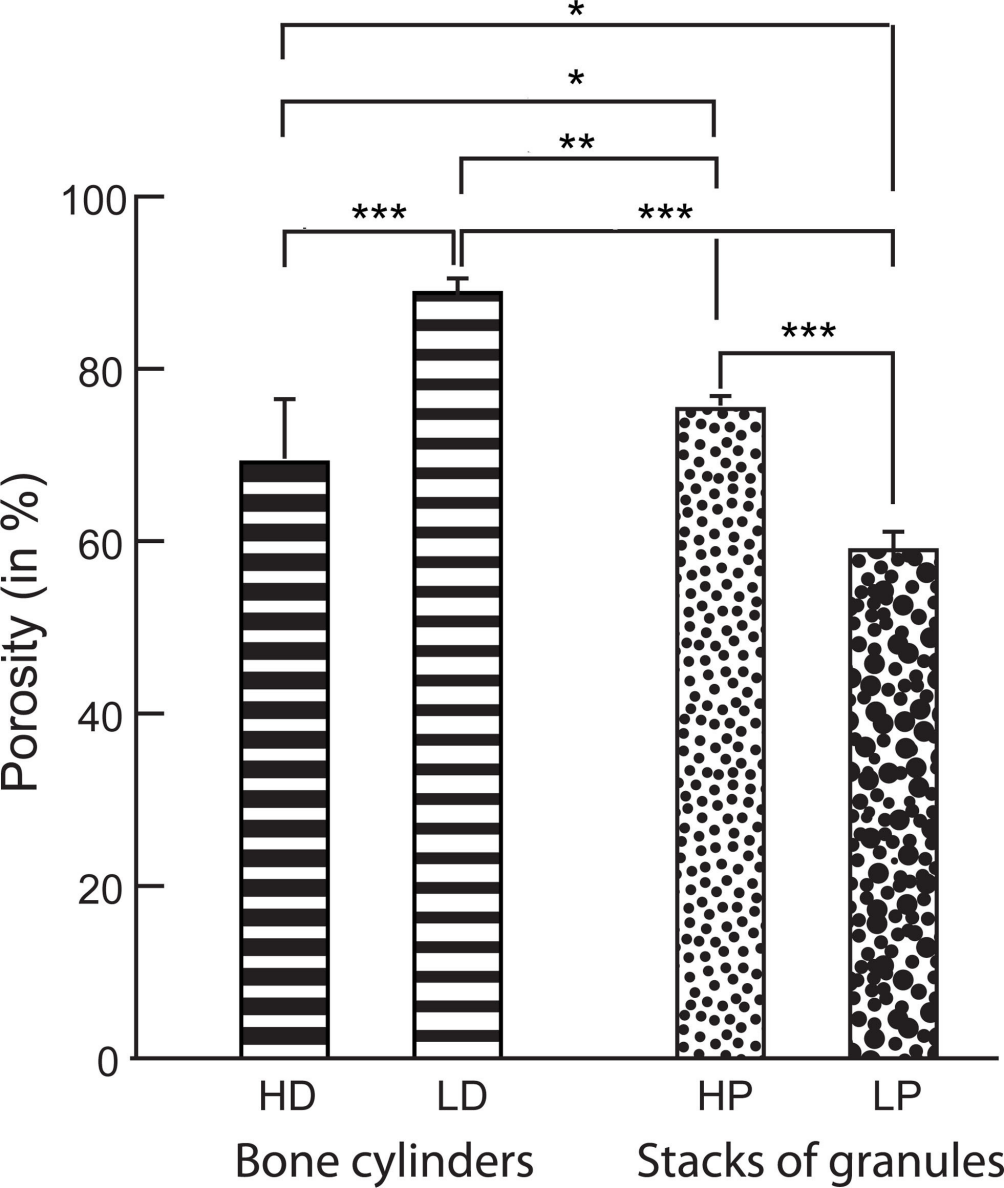
**Porosity measured by microCT in the bone cylinders and stacks of granules**. Statistical analysis was done with Kruskal–Wallis analysis of variance followed by the Conover–Inman *post hoc* test. Error bars indicate SD. *Significantly different with *p* < 0.02; **with *p* < 0.003; ***with *p* < 0.0001.

**Figure 5:**
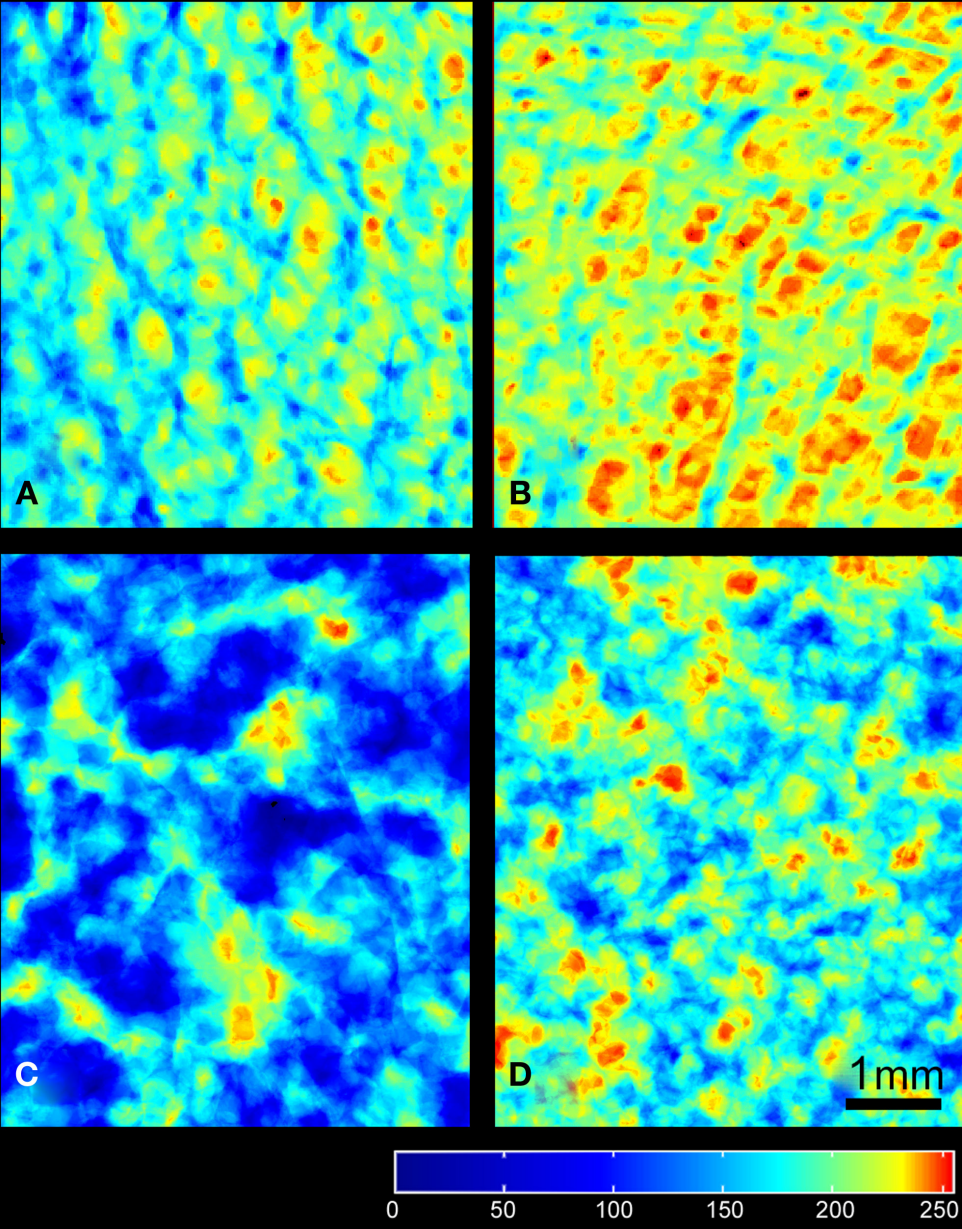
**Images of the frontal planes projected from the different types of porous materials: (A) HD bone, (B) LD bone, (C) LP, and (D) HP granules of **β**-TCP**. The regions with maximal porosity are red, minimal porosity in blue according to the LUT (at the bottom of the image). All images have been prepared with a square ROI of 5 mm side length and the scale bar represents 1 mm.

A 3D-rendering of the two types of bone cores and granule stacks is shown in Figure 3. Morphometric parameters are listed in Table 1 and illustrated in Figure 4. The HD bone cores had a significantly smaller porosity than LD bones. The LP stacks had the lowest porosity among all groups. Conversely, HP stacks presented a porosity that is intermediate between that of a HD bone and that of a low-density bone. The HP formulation produced a ~28% increase of porosity associated with a reduction of the mass of β-TCP. This was associated with a significant increase in BS/TV in the HP group compared to the LP group (+11.6%). The vector analysis of porosity clearly evidenced differences between the two pairs of materials: HD bones appeared inhomogeneous with areas containing large amounts of bony material (blue areas according to the LUT), while LD had a large number of red spots indicating HP (Figures 5A,B). Very similar findings were observed for the LP and HP stacks of granules: LP stacks had large areas of material but large holes were also present. On the contrary, the morphology of the stacks of HP granules had a more regular distribution of the pores and material (Figures 5C,D). Interestingly, the fractal dimension *D*_f_ did not differ among the four groups, but lacunarity λ was high in HD bones and even higher in the LP granules (Table 1).

## Discussion

In this series of materials comprising bone cores and granular stacks made of β-TCP granules, a quantitative and morphological analysis of porosity was done. Porosity of the LP stacks was noticeably lower than a HD trabecular bone. However, the porosity of the HP stacks (and conversely the amount of material) was in the range of normal bone volume as determined by classical histomorphometry methods (30). This reflects the microarchitecture of the stacks of HP granules which contain more macropores and thin branches than the LP granules. However, as expected, this effect is detrimental to the biomechanical properties of the HP granules whose stacks have lower compressive mechanical properties, as evidenced by lower stiffness values, lower work to failure, and maximum displacement. The surgeon should be aware not to crush the granules in the grafted areas and the use of a bone syringe with a large diameter may help.

It should be noted that the BS/TV of HP granules is significantly higher than that of LP granules or that of HD and LD bones. This is especially important since BS/BV represents the bioactive surfaces of β-TCP that will be in direct contact with the biological fluids and the cells (31). The BS/TV values obtained in this study with β-TCP granules are similar to those reported by other groups with BCP (a mixture of β-TCP and hydroxyapatite) (32). Recent observations have stressed the interest of increasing the specific surface (BS/TV) of orthophosphate materials to promote bone formation (33). So, the HP formulation represents the highest surface available for osteoblast attachment and proliferation (osteoblasts are bone-forming cells). Because β-TCP is a biomaterial colonized by osteoconduction from nearby trabeculae, this parameter is of great importance (34).

MicroCT analysis is a relatively recently introduced tool in the analysis of bone and materials. Values for parameters, such as BV/TV and Po, are highly correlated with histomorphometry on 2D sections (35). In the present study, the difference in porosity between the stacks of granules can be clearly identified as previously reported (12). Different types of parameters have been proposed to evaluate the 3D arrangement of porous materials (e.g., the box-plot fractal dimension) (22, 23) and the interconnectivity of the pores created between the granules (29). The method was successfully applied to evaluate the complexity of different types of granule aggregates: sandstone (36), coal (37), metal grains (38), and soils (39). The complexity of the granule repartition, evaluated by *D*_f_, did not differ between the four groups. However, fractal lacunarity appears as an interesting new tool capable to differentiate the groups but it does not bring morphological information (12, 28, 29, 40). We have recently developed vector analysis for visualization and quantification of the porosity in objects presenting a complex form (19). The method was found useful to analyze pores in materials prepared with various types of porogens and in bones having a particularly complex shape. This is the case with alveolar bone at the mandible, which is very difficult to analyze because of the presence of the tooth roots. Recently, the bone loss induced by paralysis of *Mus masseter* and *Mus temporalis* was evidenced by this technique (41). The frontal plane is produced line by line during the vector analysis of each microCT image and represents the projection of porosity. The complexity of the image can be measured by fractal geometry on this projected 2D image. In the present series, there was no difference for *D*_f_ among the four groups under study. However, lacunarity is known to be influenced by the uniformity of the spatial distribution; it is a useful parameter when objects do not differ by their fractal dimension (42, 43). The maximal λ value was reached in the LP (25 g) group meaning that very large pores are created in the 3D arrangement of the granules. On the other hand, the lowest λ values were obtained for the HP (12.5 g group) granules and the LD bone, which presented a regular distribution of pore dimensions. Similar relationships have been reported in studies concerned with the porosity of soils (44, 45).

The presence of pores larger than 100 μm has been recognized as a key factor in the vascular invasion of grafted materials into bone (46, 47). In this study, Po⋅Diam values for bone were within the values typically reported for bone (48); Po⋅Diam of both HP and LP are larger than 300 μm and well suited for body fluid and cell invasion (49).

There are some limitations to this study: (1) no compressive analysis was done on the bone cylinders, but synthetic ceramics are well known to be more brittle than bone; (2) bovine bone was used to obtain the HD cylinders, but the biomechanical parameters for trabecular bone are very close (stiffness in compression for bovine bone: 173 MPa vs. human: 139–472 MPa) (50); and (3) the other microarchitectural characteristics of the β-TCP materials having been reported elsewhere are not duplicated in the present study (12).

## Conclusion

The present study shows that the two types of β-TCP granules prepared differently did not produce the same interconnected porosity: LP granules provided very large but less numerous pores and HP granules provided a HP with a large interface. The HP stacks of granules presented a porosity similar to trabecular bone, although the granules were physically independent. This study confirms that vector analysis is a suitable method for the evaluation of porosity of complex objects.

## Conflict of Interest Statement

The authors declare that this research was conducted in the absence of any commercial or financial relationships that could be construed as a potential conflict of interest.
